# Octahedral Tin Dioxide Nanocrystals Anchored on Vertically Aligned Carbon Aerogels as High Capacity Anode Materials for Lithium-Ion Batteries

**DOI:** 10.1038/srep31496

**Published:** 2016-08-11

**Authors:** Mingkai Liu, Yuqing Liu, Yuting Zhang, Yiliao Li, Peng Zhang, Yan Yan, Tianxi Liu

**Affiliations:** 1 School of Chemistry and Chemical Engineering, Jiangsu Key Laboratory of Green Synthetic Chemistry for Functional Materials, Jiangsu Normal University, Xuzhou 221116, China; 2 State Key Laboratory of Molecular Engineering of Polymers, Department of Macromolecular Science, Fudan University, Shanghai 200433, China; 3 State Key Laboratory for Modification of Chemical Fibers and Polymer Materials, College of Materials Science and Engineering, Donghua University, Shanghai 201620, China

## Abstract

A novel binder-free graphene - carbon nanotubes - SnO_2_ (GCNT-SnO_2_) aerogel with vertically aligned pores was prepared via a simple and efficient directional freezing method. SnO_2_ octahedrons exposed of {221} high energy facets were uniformly distributed and tightly anchored on multidimensional graphene/carbon nanotube (GCNT) composites. Vertically aligned pores can effectively prevent the emersion of “closed” pores which cannot load the active SnO_2_ nanoparticles, further ensure quick immersion of electrolyte throughout the aerogel, and can largely shorten the transport distance between lithium ions and active sites of SnO_2_. Especially, excellent electrical conductivity of GCNT-SnO_2_ aerogel was achieved as a result of good interconnected networks of graphene and CNTs. Furthermore, meso- and macroporous structures with large surface area created by the vertically aligned pores can provide great benefit to the favorable transport kinetics for both lithium ion and electrons and afford sufficient space for volume expansion of SnO_2_. Due to the well-designed architecture of GCNT-SnO_2_ aerogel, a high specific capacity of 1190 mAh/g with good long-term cycling stability up to 1000 times was achieved. This work provides a promising strategy for preparing free-standing and binder-free active electrode materials with high performance for lithium ion batteries and other energy storage devices.

Lithium-ion batteries (LIBs), as one of the most important energy-storage devices, have attracted tremendous attentions from both scientific and industrial fields due to their high energy density, low self-discharge, and environmental friendliness^1^,^2^. Developing new electrode materials with ultrahigh specific capacity and good cycling stability for LIBs is a crucial step to promote their large scale applications in energy storage units^3^,^4^,^5^. Up to now, a great number of interests have been generated to develop high-power anode materials with various nanostructures and morphologies to facilitate the next generation of high-performance rechargeable LIBs^6^,^7^,^8^,^9^. Among numerous anode materials including metal, metal oxide/dioxide and conjugated polymers, tin dioxide (SnO_2_) is considered as one of the most important active anode materials for energy storage due to their high theoretical capacity, low potential of lithium ion intercalation, no toxicity and low cost features^10^,^11^,^12^,^13^,^14^,^15^,^16^. Especially, SnO_2_ octahedral nanocrystals exposed to high-energy facets exhibit much enhanced lithium ions storage ability compared with the irregular SnO_2_ nanoparticles exposed to stable facets^17^. The remarkably improved electrochemical performance of SnO_2_ octahedral nanocrystals in LIBs can be ascribed to the reason that high-energy facets have an open surface structure and possess a high density of atomic sections and edges, coupled with a large number of unsaturated coordination sites for lithium ions insertion/extraction^18^.

However, SnO_2_ octahedral nanocrystals encounter similar disadvantages of poor recyclability with the common SnO_2_ nanomaterials due to their drastic volume expansion/shrinkage during the alloying reaction with lithium ions^19^,^20^. This phenomenon is believed to be the result of the pulverization of active materials, which can further block the electrical contact pathways between adjacent particles and fatigue failure and disintegration of the active SnO_2_-based electrode materials^21^,^22^. Hybridizing SnO_2_ nanomaterials with carbon nanomaterials, especially carbon nanotubes (CNTs) and graphene sheets, and nanostructured engineering of SnO_2_ with various morphologies seem to be an effective way to improve their electrochemical performance^20^,^23^. Particularly, many groups have developed graphene-SnO_2_ electrodes for LIBs with promising electrochemical performance due to the excellent electronic conductivity and superior mechanical flexibility of graphene sheets^24^,^25^. However, hybridizing SnO_2_ with graphene or CNTs by a simple mixing method cannot realize uniform distribution of SnO_2_ nanoparticles on the carbonic matrix due to their high surface energy, and cannot afford efficient space for volume expansion of SnO_2_. To overcome these problems, fabricating sandwich-structured graphene-SnO_2_ nanomaterials with good porous structures and excellent dispersion of SnO_2_ nanoparticles seems to be an effective method to exploit the superior performance of SnO_2_, because good distribution of SnO_2_ nanoparticles can achieve the full utilization of their active sites, and the porous graphene matrix can accelerate the electron transport, as well as provide sufficient expansion volume for the lithiation of SnO_2_. Thus, developing a versatile method for preparing three dimensional (3D) porous SnO_2_-based active materials by hybridizing unique SnO_2_ octahedrons with excellent conductive matrix with excellent distribution is of great importance for the promotion of active electrode materials based on metal oxide or other highly active materials for energy storage applications.

In addition, binder and additional carbon fillers were widely used for preparing active electrodes for LIBs, with the objective of pasting the active materials on the collectors and accelerating the transport kinetics of electrons inside the electrodes^26^,^27^. However, binder materials (e.g. polyvinylidene fluoride, PVDF) have not any ability for storing lithium ion, but damage the conductive networks of the active materials. Additional carbon fillers with barely any lithium ion storage ability were mass employed (10–20 wt%), resulting in the decreasing of the energy density and specific capacity of assembled LIBs. Therefore, developing high active electrodes without any utilization of binder and additional carbon fillers is of great importance for the solid progress of LIB scientific research systems.

In this work, we report a simple and effective strategy to fabricate 3D giant graphene sheets-carbon nanotubes (GCNT)-SnO_2_ octahedrons (GCNT-SnO_2_) aerogels, in which octahedral SnO_2_ nanoparticles exposed of high-energy {221} facets were tightly anchored on the surface of GCNT. Importantly, there are vertically aligned pores inside the GCNT-SnO_2_ aerogels which can efficiently prevent the emersion of “closed” pores, but can provide sufficient expansion space for SnO_2_ octahedrons during the long-term cycling. Meanwhile, the high-energy facets of SnO_2_ can be fully exposed to the lithium ions due to their perfect interfacial distribution on GCNT matrix. Benefiting from the good immersion of electrolyte, superior electrical conductivity of GCNT matrix, full utilization of SnO_2_ octahedrons, and the synergistic effect of GCNT and SnO_2_, the resultant GCNT-SnO_2_ aerogels achieve a rapid insertion/extraction of lithium ions (as illustrated in Fig. 1), and exhibit an ultrahigh specific capacity (up to 1190 mAh/g), excellent rate capability, as well as highly reversible capacity (80% retention after 1000 cycles), demonstrating their great potential prospects as electrode materials for LIBs.

## Results and Discussion

**Figure 2:**
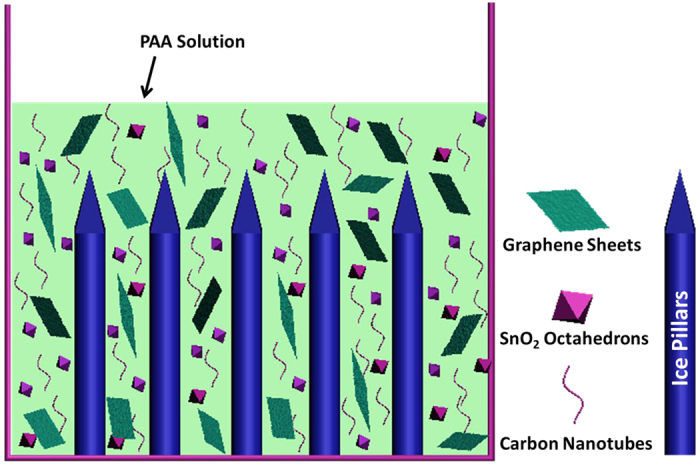
Vertically aligned pores produced with the assistance of ice pillars created in the directional freezing treatment.

The preparation process for GCNT-SnO_2_ aerogel with aligned pores is schematically illustrated in Figure S1. Herein, pristine CNTs with bundle morphology can be homogeneously dispersed by graphene oxide sheets under strong sonication, according to the intense interfacial interactions including van der Waals and π-π stacking^28^. The prepared SnO_2_ octahedrons can be uniformly dispersed on the surface of CNT/graphene oxide composite with the assistance of 2-[2-(2-Methoxyethoxy)ethoxy]acetic acid (MEEAA). Low weight percent of poly(amic acid) (PAA) (0.5 wt%) was introduced in the CNT/graphene oxide/SnO_2_ composite solution in order to induce their aligned arrangement during directional freezing process. The vertically aligned pores were produced by the vertically aligned ice pillars formed in the directional freezing process (Fig. 2), following by the treatment of freeze-drying and high temperature pyrolysis.

**Figure 3:**
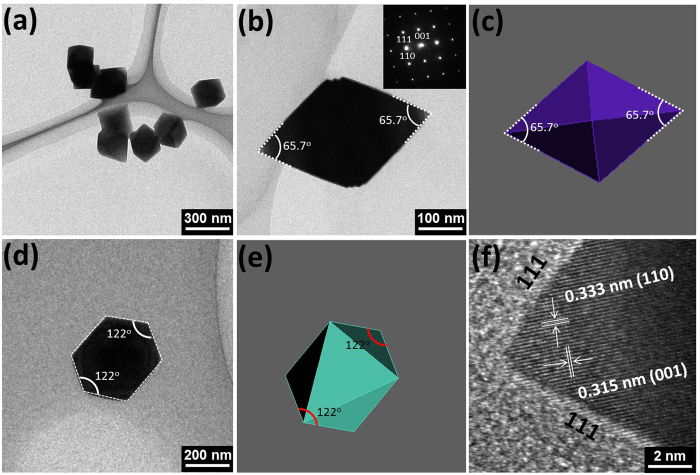
TEM images of prepared samples. (**a**) TEM image of SnO_2_ octahedrons. (**b**) low-magnification TEM image of a SnO_2_ octahedron viewed along the {110} direction (inset shows the corresponding SEAD pattern) with (**c**) its schematic model enclosed by {221} facets. (**d**) TEM image of the same SnO_2_ octahedron projected in the {111} direction with (**e**) its schematic model. (**f** ) HRTEM image taken from the top apex of SnO_2_ octahedron enclosed by {221} facets.

Detailed structural information of SnO_2_ octahedrons is provided by the transmission electron microscopy (TEM) images and selected-area electron diffraction (SAED), as seen in Fig. 3. TEM image of several SnO_2_ octahedrons (Fig. 3a) with random configuration indicates the uniform size of prepared octahedrons. Figure 3b shows the TEM image of single octahedron projected along the {110} direction with corresponding SAED pattern (inset). The single-crystalline characteristics of SnO_2_ octahedrons can be indexed by the {110} zone axis in the SAED pattern^29^. Schematic model of octahedron (Fig. 3c) enclosed by {221} facets exhibits the same apex angle of 65.7^o^ as that of SnO_2_ particle in Fig. 3b. The same SnO_2_ particle was rotated to the {111} zone axis (Fig. 3d), and both the outline and the apex angle of the particle still corresponded well with the octahedral model enclosed by {221} facets (Fig. 3e). High-resolution TEM (HRTEM) image taken from the top apex of SnO_2_ octahedron exhibits lattice fringes of 0.333 and 0.315 nm, corresponding to the {110} and {001} planes of SnO_2_ octahedron. Based on these TEM observations and structural analysis, it can be concluded that the as-prepared SnO_2_ particles are exposed with the {221} high-energy facets with uniform size.

**Figure 4:**
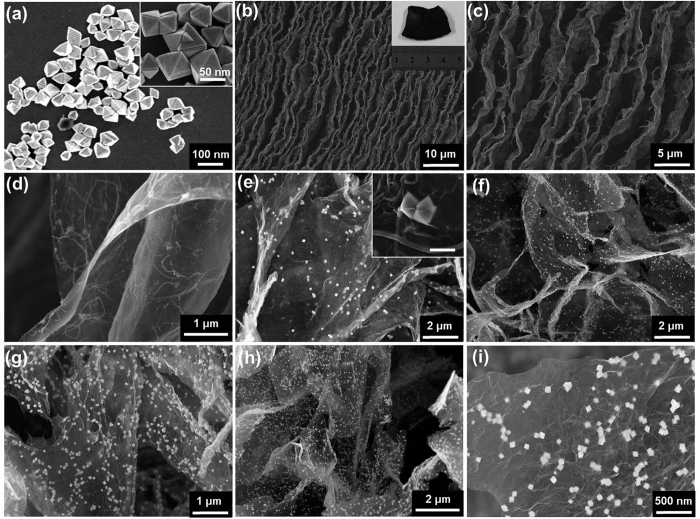
SEM images of prepared samples. (**a**) Pure SnO_2_ octahedrons at low and high (inset) magnifications. (**b,c**) GCNT-SnO_2_ aerogels with vertically aligned pores at different magnifications, and the inset in (**b**) shows the optical image of GCNT-SnO_2_ aerogel. (**d**) CNTs tightly bonded on the surface of giant graphene sheets. (**e**) GCNT-SnO_2_(1) aerogels with low content of SnO_2_ octahedrons, and the inset confirms the clear octahedron-shaped morphology of SnO_2_ octahedrons in GCNT-SnO_2_ aerogel. (**f–h**) SEM images of GCNT-SnO_2_(2), GCNT-SnO_2_(3) and GCNT-SnO_2_(4) aerogels, respectively. (**i**) High resolution SEM image of enlarged part of GCNT-SnO_2_(4) aerogel.

The morphology of SnO_2_ octahedrons and G/CNT-SnO_2_ aerogels were characterized by scanning electron microscopy (SEM), as seen in Fig. 4. Figure 4a shows that the SnO_2_ octahedrons consist of high-purity particles with smooth surfaces and edges, and the inset image confirms the well-defined octahedron-shaped morphology of the obtained SnO_2_ particles. Figure 4b,c present the GCNT-SnO_2_ aerogel with vertically aligned pores at low and high magnifications. These aligned pores can effectively connect the holes inside GCNT-SnO_2_ aerogel, and further prevent the emergence of “closed” pores. The vertically aligned pores can ensure the thorough immersion of electrolyte but can also expose all their porous structures and active sites to lithium ions contained in electrolyte. Inset in Fig. 4b exhibits the optical image of GCNT-SnO_2_ aerogel with free-standing architecture. Figure 4d shows the composite of CNTs and graphene sheets. It can be seen that the CNTs are thoroughly dispersed and tightly bonded on the surface of graphene sheets. Interestingly, the introduced CNTs on the surface of graphene sheets can act as skeletons between different graphene sheets to greatly decrease their tightly interfacial stacking. Furthermore, CNTs up to several micrometers in length can bridge different graphene sheets as a connecting conductive pathway. SEM image of GCNT-SnO_2_(1) aerogel was presented in Fig. 4e, and the SnO_2_ particles were homogenously dispersed on the surface of GCNT composite without any aggregation. The good dispersion of SnO_2_ octahedrons and their perfect interfacial contacting with GCNT, coupling with the good permeability of vertically aligned pores, can achieve excellent synergistic effect in lithium ion storage application. Figure 4f–h present the SEM images of GCNT-SnO_2_(2), GCNT-SnO_2_(3), and GCNT-SnO_2_(4) aerogels, respectively, which were prepared by increasing the amount of SnO_2_ octahedrons by 2, 3, and 4 times in the resulted GCNT-SnO_2_ aerogels. Interestingly, the good interfacial distribution of SnO_2_ octahedrons on GCNT surface was not affected by their increased content, even up to four times weight of GCNT, as seen in the enlarged picture of GCNT-SnO_2_(4) aerogel at high magnification (Fig. 4i). TEM images were used to further analyze the morphology of GCNT-SnO_2_ aerogels (Figure S2), and no aggregation was observed in both GCNT-SnO_2_(1) and GCNT-SnO_2_(3) samples. Especially, several SnO_2_ octahedrons with ambiguous edges or frames due to the coverage effect of graphene sheets confirm that the SnO_2_ particles were deposited on both sides of GCNT composite sheets. The good dispersion of SnO_2_ on GCNT can be further confirmed by the Energy disperse spectroscopy (EDS) mapping detection, as seen in Figure S3. Sn (Figure S3c) and O (Figure S3d) elements can be distinguished as observed on the carbon layer (Figure S3b), agreeing well with the SEM image of GCNT-SnO_2_(3) aerogel (Figure S3a). The weight percent of SnO_2_ in GCNT-SnO_2_(1), GCNT-SnO_2_(2), GCNT-SnO_2_(3) and GCNT-SnO_2_(4) aerogels are about 43%, 57%, 72% and 80%, respectively, which were tested by thermogravimetric analysis (TGA) (Figure S4). These results verified the rational and credible design of SnO_2_/GCNT ratio in GCNT-SnO_2_ aerogels.

**Figure 5:**
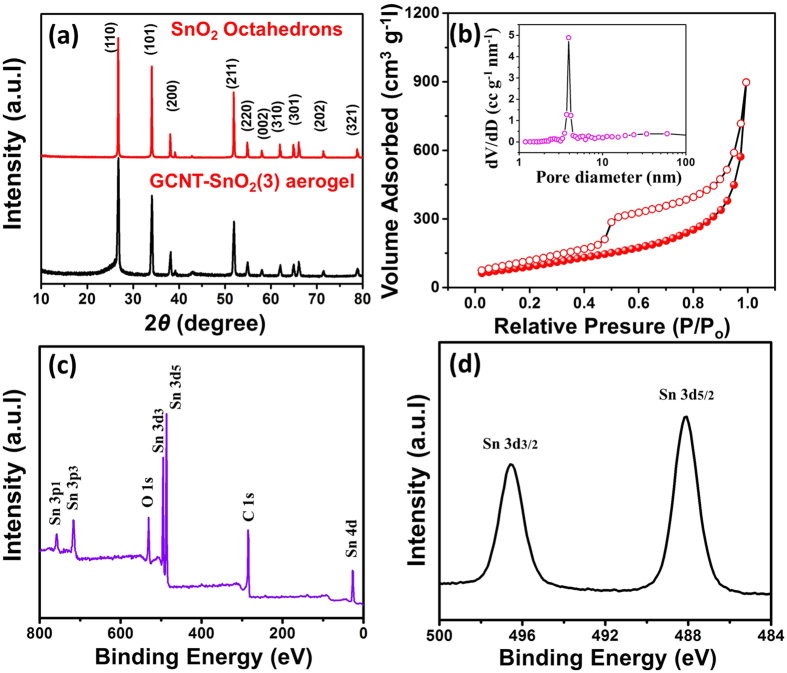
XRD, BET and XPS observations of as-prepared samples. (**a**) XRD patterns of pure SnO_2_ octahedrons and GCNT-SnO_2_(3) aerogel, (**b**) Nitrogen adsorption/desorption isotherm and pore size distribution of GCNT-SnO_2_(3) aerogel observed at 77 K, (**c**) XPS survey spectra and (**d**) Sn 3d spectra of GCNT-SnO_2_(3) aerogel.

The crystalline structures of SnO_2_ octahedrons and GCNT-SnO_2_(3) aerogels were investigated by X-ray diffraction (XRD), as seen in Fig. 5a. For pure SnO_2_ octahedrons, all the peaks can be readily indexed to the rutile phase SnO_2_ (JCPDS no. 41-1445)^30^,^31^. The XRD pattern of GCNT-SnO_2_(3) aerogel shows similar diffraction peaks with the SnO_2_ octahedrons, indicating the crystalline morphology of SnO_2_ was not eroded after the introduction of GCNT matrix. The appearance of a broadened peak at 2θ = 26.1° corresponding to the (002) of graphite indicates the existence of graphene and CNTs. The vertically aligned pores inside GCNT-SnO_2_ aerogels coupled with the good distribution of SnO_2_ particles can positively contribute to the increase of their specific surface area. Figure 5b shows the nitrogen isothermal adsorption/desorption result and the corresponding pore size distribution of GCNT-SnO_2_(3) aerogel. High BET surface area of 344 m^2^/g was observed, which is much larger than 34 m^2^/g of pure SnO_2_ octahedrons (Figure S5). In addition, based on the Barrett-Joyner-Halenda (BJH) model (inset in Fig. 5b), the pore size of GCNT-SnO_2_(3) aerogel is centered at ~4 nm. The greatly enhanced surface area of GCNT-SnO_2_(3) aerogel associated with the meso- and macroporous structures is favorable for the electrolyte accessibility and fast lithium ion diffusion^32^,^33^. To further confirm the chemical compositions of GCNT-SnO_2_ aerogel, X-ray photoelectron spectroscopy (XPS) measurements were performed on GCNT-SnO_2_(3) aerogel in the range of 0 - 800 eV, as seen in Fig. 5c. The peaks located at the C, O, and Sn core level regions can be assigned as C 1 s, O 1 s, Sn 3p, Sn 3d, and Sn 4d, respectively. Two peaks centered at 496.9 and 488.0 eV can be attributed to the Sn 3d_3/2_ and Sn 3d_5/2_ (Fig. 5d)^34^,^35^, and the barely detected C = O and C-O-C peaks in the C 1 s region (Figure S6) confirm the good chemical reduction effect of hydrazine vapor. Moreover, sheet resistance of prepared samples was detected based on a four-probe method. As seen in Table S1, GCNT-SnO_2_ aerogels exhibit low sheet resistance from 79.4 to 105.9 Ω sq^−1^, which is comparable with the ITO and commonly used graphene sheets^36^,^37^.

**Figure 6:**
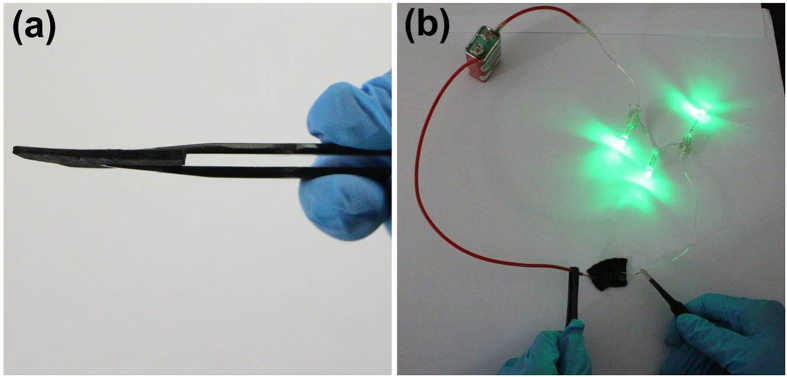
Morphology and conductive ability of GCNT-SnO_2_(3). (**a**) Film architecture of GCNT-SnO_2_(3) aerogels, and (**b**) closed circuit with GCNT-SnO_2_ aerogels replacing the copper wire.

The free-standing GCNT-SnO_2_(3) aerogel with film architecture (Fig. 6a) ensures them to be directly used without any binder or additional carbon fillers. Interestingly, GCNT-SnO_2_ aerogel can be used in a closed circuit as a substitution of copper wire (Fig. 6b), and the high brightness of green light emitting diodes (LEDs) confirms the good electrical conductivity of the prepared GCNT-SnO_2_ aerogels. These results permit the GCNT-SnO_2_ aerogels to be utilized as promising candidate as electrode materials in LIBs.

**Figure 7:**
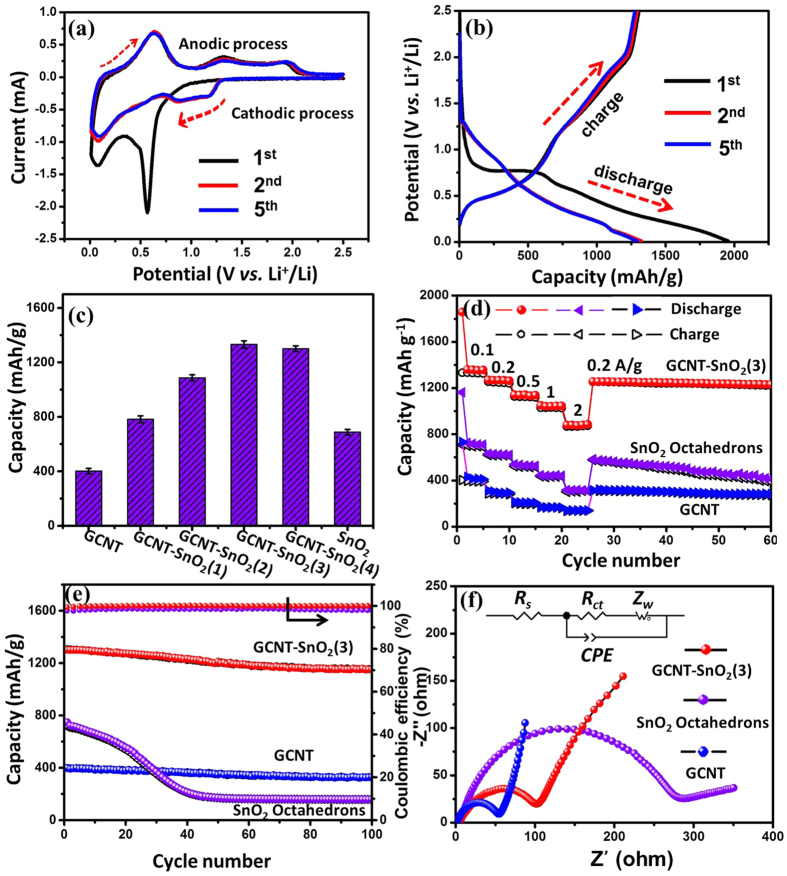
Electrochemical performance of GCNT-SnO_2_ aerogels compared with GCNT and pure SnO_2_ octahedrons. (**a**) CV curves at 0.1 mV/s and (**b**) charge/discharge curves at 0.1 A/g at the 1^st^, 2^nd^, and 5^th^ cycle of GCNT-SnO_2_(3) aerogel electrode. (**c**) Specific capacity of GCNT, GCNT-SnO_2_ aerogels and pure SnO_2_ octahedrons calculated from the 5^th^ discharge curves at 0.1 A/g. (**d**) Rate performance of GCNT, GCNT-SnO_2_(3) aerogels and pure SnO_2_ octahedrons at various current rates from 0.1 to 2 A/g. (**e**) Cycling stability at 0.1 C coupled with corresponding Coulombic efficiency and (**f** ) Nyquist plots (inset: equivalent circuit mode) of GCNT, GCNT-SnO_2_(3) aerogels and pure SnO_2_.

The electrochemical performance of GCNT, GCNT-SnO_2_ aerogels and pure SnO_2_ octahedrons acting as electrode materials for LIBs was investigated. Figure 7a shows the typical cyclic voltammogram (CV) curves of GCNT-SnO_2_(3) aerogel as electrode materials for LIBs over a voltage range of 0.01~2.5 V *vs*. Li/Li^+^. In the first cycle, an irreversible reduction peak with a maximum value at 0.57 V was emerged, which can be attributed to the formation of a solid electrolyte interface (SEI) layer, as well as the reduction of SnO_2_ to amorphous lithium oxide and metallic Sn (equation 1)^38^. The cathodic peak closed to 0 V can be attributed to the lithium alloying reaction with Sn (equation 2), and the oxidation peak at 0.6 V for all the following cycles can be ascribed to the corresponding dealloying reaction^39^. The oxidation peaks observed at 1.30 V and 1.84 V are resulted from the partially reversible reactions of formation of SEI layer and SnO_2_^40^. In addition, an obvious oxidation peak around 0.14 V in the anodic process represents the lithium extraction from GCNT matrix (equation 3). Compared with the indistinctive oxidation/reduction peaks of GCNT (Figure S7a), GCNT-SnO_2_ aerogels with other mass ratios exhibit apparent oxidation/reduction peaks (Figure S7b–S7e) as that of SnO_2_ octahedrons, confirming the efficient incorporation of SnO_2_ with the GCNT matrix. Sample GCNT-SnO_2_(3) aerogel exhibits the largest anodic/cathodic current density compared with the others, indicating its largest specific capacity as a result of the synergistic effect of conductive GCNT matrix and SnO_2_ octahedrons exposed of high-energy facets. Interestingly, CV curves of GCNT-SnO_2_(3) aerogel up to 60 cycles (Figure S7f) exhibit similar oxidation/reduction peaks with nearly undiminished current intensity as before, which demonstrates its good endurance property upon long term cycling.


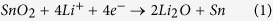



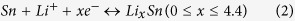



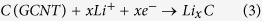


Figure 7b shows the charge/discharge curves of GCNT-SnO_2_(3) aerogel on the 1^st^, 2^nd^, and 5^th^ cycles at a rate of 0.1 A/g. Voltage plateaus observed on the charge/discharge curves corresponding to the oxidation/reduction peaks in the CV curves can be ascribed to the lithium ion insertion/extraction reactions. Comparatively, sample GCNT does not show any potential plateaus during the charge/discharge process (Figure S8a), as a result of the different insertion mechanism of lithium ions^41^. GCNT-SnO_2_(3) aerogel gives a much higher discharge capacity of 1750 mAh/g compared with 710 mAh/g of GCNT and 1060 mAh/g of pure SnO_2_ octahedrons (Figure S8e) in the first discharge curve, with the corresponding Coulombic efficiencies of 68%, 57.8% and 54.5%, respectively. Similarly, samples of GCNT-SnO_2_(1), GCNT-SnO_2_(2) and GCNT-SnO_2_(4) aerogels (Figure S8b–S8d) exhibiting alike lithium ion insertion/extraction voltage plateaus as that of GCNT-SnO_2_(3) aerogel also undergo conspicuous capacity loss during the first charge/discharge cycle. The huge capacity loss of the prepared samples can be ascribed to the irreversibility resulting from SnO_2_ reduction and the formation of SEI layer on the surface of active materials^42^,^43^. Charge/discharge curves from the 6^th^ to 100^th^ cycles of GCNT-SnO_2_(3) aerogel were recorded, as seen in Figure S9f. The insignificant decline upon the 100 cycles indeed confirms the good cycling stability of GCNT-SnO_2_(3) aerogel. Specific capacities of the prepared samples calculated from the discharge curves on the 5^th^ cycle were compared (Fig. 7c). GCNT-SnO_2_(1), GCNT-SnO_2_(2), GCNT-SnO_2_(3), GCNT-SnO_2_(4) aerogels exhibit greatly enhanced specific capacity of 720, 1027, 1190 and 1034 mAh/g, respectively, compared to 402 mAh/g of GCNT and 688 mAh/g of pure SnO_2_ octahedrons, due to the synergistic effect of GCNT and SnO_2_ for lithium ion storage. Here, the GCNT-SnO_2_(4) aerogel with higher content of SnO_2_ shows a little lower specific capacity compared with GCNT-SnO_2_(3), which may be resulted from the excessive loading of SnO_2_ octahedrons that the utilization of active sites of SnO_2_ was not so effectively as before.

The rate capabilities of GCNT-SnO_2_ aerogels compared with GCNT and SnO_2_ octahedrons were also investigated from current densities from 0.1 to 2 A/g, as seen in Fig. 7d. The GCNT-SnO_2_(3) aerogel displays excellent rate capabilities and delivers rate capacities of 1190, 1095, 974, 875, 735 mAh/g at current densities of 0.1, 0.2, 0.5, 1, and 2 A/g, respectively. Clearly, GCNT-SnO_2_(3) aerogel exhibits much higher capacity compared with GCNT (165 mAh/g) and pure SnO_2_ octahedrons (296 mAh/g) at high current density of 2 A/g. It should be noted that, GCNT-SnO_2_(3) aerogel delivers a comparable specific capacity of 1143 mAh/g as before when the current density returns to 0.1 A/g, and also exhibits good cycling performance in the following 35 cycles. In addition, GCNT-SnO_2_(1), GCNT-SnO_2_(2) and GCNT-SnO_2_(4) aerogels also exhibit good rate performances under different current densities, as seen in Figure S9. The superior rate performance of GCNT-SnO_2_ aerogels clearly demonstrates that the successful hybridization of SnO_2_ octahedrons on GCNT matrix endows them with perfect tolerance to varied discharge current densities, and good prospect in high power LIBs. The excellent rate capabilities of GCNT-SnO_2_ aerogels were probably rooted in high energy facets exposed by SnO_2_ octahedrons, coupling with the good distribution of SnO_2_ on the conductive GCNT matrix, as well as the highly porous structures of GCNT-SnO_2_ aerogels.

Figure 7e shows the relative cyclic performance of the GCNT-SnO_2_(3) aerogel, GCNT and SnO_2_ octahedrons at 0.1 A/g. SnO_2_ octahedrons exhibit a high specific capacity of 688 mAh/g, but possess a very poor recyclability with dramatic decrease of the capacity to 175 mAh/g only after 40 cycles. This poor cycling performance of pure SnO_2_ octahedrons was caused by the large volume expansion taking place during the rapid lithium ion insertion/extraction process, which further deteriorates the intimate contact between active SnO_2_ particles and the current collector. GCNT material displays an excellent cycling stability upon 100 cycles, but exhibits a low specific capacity of 404 mAh/g, due to lack of sufficient active sites. GCNT-SnO_2_(3) aerogel exhibits good cycling stability with a capacity retention of 83% after 100 cycles, and also possesses a high Coulombic efficiency up to 99% after the first five cycles. GCNT-SnO_2_ aerogels with other contents of SnO_2_ also exhibit good cycling performance and high Coulombic efficiency under a current density of 0.1 A/g, as seen in Figure S10. The much better cycling stability of GCNT-SnO_2_ aerogels compared to the result of pure SnO_2_ octahedrons was offered by the synergistic effect from the efficient combination strategy. Particularly, SnO_2_ octahedrons with high-energy facets provide sufficient active sites for lithium ions, good interfacial contact between GCNT and SnO_2_ particles offers excellent transport of electrons, and the vertically aligned pores inside GCNT-SnO_2_ aerogels ensure thorough immersion of electrolyte throughout the electrodes. Moreover, GCNT-SnO_2_ aerogels can be directly used as electrode materials without any binder, conductive additives or current collectors, which further contributes to their superior electrochemical performance. In addition, the morphologies of GCNT-SnO_2_(3) sample after the 1^st^ cycle (Figure S11) and the 100^th^ cycles (Figure 12) were provided. As can be seen, the octahedron morphology of SnO_2_ was integrally maintained after the 1^st^ cycle. After 100 cycles, the SnO_2_ nanoparticles are still homogeneously dispersed on the surface of GCNT, but the octahedron morphology of SnO_2_ becomes ambiguous (Figure S12a,S12b), which is consistent with the TEM observation of GCNT-SnO_2_(3) (Figure S12c). HRTEM image of single SnO_2_ octahedron separated from the GCNT-SnO_2_(3) aerogel after 100 cycles confirms its good interplanar spacing, which can further confirm the good cycling stability of the GCNT-SnO_2_(3) aerogel.

Electrochemical impedance spectra (EIS) of the prepared samples were recorded in order to deeply understand their different performances as electrodes in LIBs. Figure 7f shows the EIS curves of GCNT, GCNT-SnO_2_(3) aerogel and pure SnO_2_ octahedrons. Typically, each of these three EIS curves exhibits a semicircle in the high frequency range and a sloping straight line in the low frequency range. EIS curve of GCNT-SnO_2_(3) aerogel, coupling with the results of GCNT-SnO_2_(1), GCNT-SnO_2_(2) and GCNT-SnO_2_(4) aerogels (Figure S13), show much smaller radii than that of pure SnO_2_ octahedrons. Solution resistance (*R*_s_) and Warburg impedance (*Z*_w_) of these electrodes were also recorded according to the equivalent circuit (inset in Fig. 7f). Resistance values calculated based on the equivalent circuit were listed in Table S2. *R*_ct_ values of GCNT-SnO_2_ aerogels (101.3~119.5 Ω) were greatly decreased compared with the result (264.8 Ω) of pure SnO_2_ octahedrons, confirming that the charge transfer resistance of GCNT-SnO_2_ aerogel electrode was greatly decreased with the assistance of GCNT conductive matrix. EIS curves of GCNT-SnO_2_(3) aerogel electrode after the 1^st^ cycle and the 100^th^ cycle were recorded, as seen in Figure S14. The two impedance spectra exhibit similar semicircle shape in high frequency and straight line in low frequency range, indicating their excellent stability of interfacial transfer of ions and electrons. And the slightly decreased *R*_s_ and *R*_ct_ values (inset in Figure S14) of GCNT-SnO_2_(3) aerogel electrode after the 100^th^ cycle compared with the values after the 1^st^ cycle can be ascribed to the activation effect of the multiple cycles of charge/discharge.

**Figure 8:**
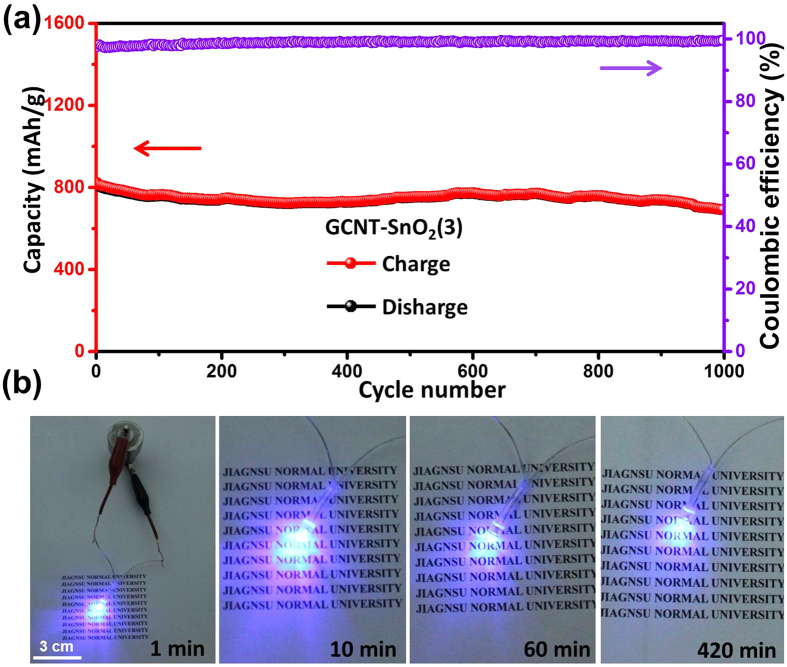
Electrochemical performance and practical application of LIBs based on GCNT-SnO_2_(3). (**a**) Ultra-long term cycling stability and Coulombic efficiency of LIB based on GCNT-SnO_2_(3) at 2 C, and the battery was initially run at 0.1 C for two cycles. (**b**) A purple LED lit up by the assembled LIB up to 420 min.

**Figure 9:**
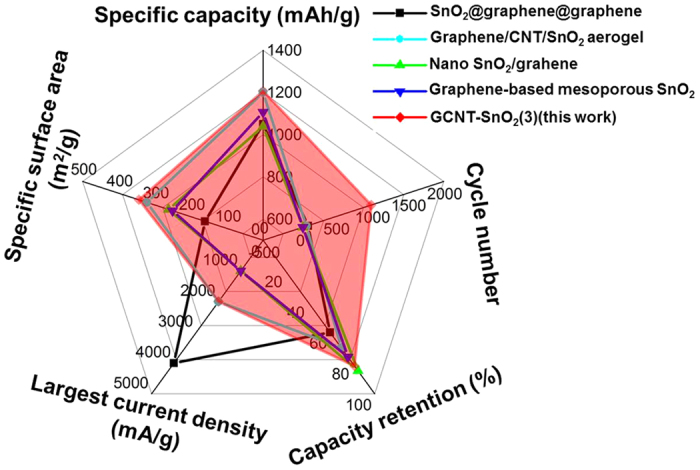
Comparison on electrochemical performances of this GCNT-SnO_2_(3) aerogel with previously reported SnO_2_ based active materials.

Interestingly, with GCNT-SnO_2_(3) aerogel as working electrode without any binder and conductive additives, the assembled lithium ion battery can be steadily cycled for 1000 cycles at 2 A/g, achieving a promising capacity retention of 80% (Fig. 8a). The assembled batteries with GCNT-SnO_2_(3) aerogel electrodes can be successfully used to light up the LED light up to 420 min (Fig. 8b). Furthermore, due to the well-designed architectural morphologies, especially the vertically aligned pores throughout the aerogels, the prepared GCNT-SnO_2_(3) aerogel acting as anode material for LIBs without any binders or conductive additives exhibits comparable or much higher electrochemical properties with other SnO_2_ based active materials^30^,^25^,^26^,^39^, as seen in Fig. 9.

## Conclusions

In summary, a novel GCNT-SnO_2_ aerogel film with vertically aligned pores was prepared by integrating SnO_2_ octahedrons and multidimensional carbon nanomaterials with a directional freezing method. The designed architecture shows excellent electrochemical performance with the largest specific capacity of 1190 mAh/g, as well as long-term cycling stability up to 1000 times. Of more importance, the vertically aligned pores can effectively prevent the emersion of “closed” pores which cannot load the active SnO_2_ nanoparticles, further ensure adequate immersion of electrolyte throughout the aerogel, and largely shorten the transport distance between lithium ion and active sites of SnO_2_. Especially, vertically aligned pores inside GCNT-SnO_2_ aerogel create meso- and macroporous structures with large surface area and excellent electrical conductivity, achieving great benefit to the favorable transport kinetics for both lithium ion and electrons. Any binder or additional carbon fillers were not employed in the GCNT-SnO_2_ aerogel electrode that greatly simplifies the electrode preparation process, and on the other hand, efficiently enhances the energy density and specific capacity of the prepared electrodes. Therefore, this work provides a general and effective approach to prepare active electrodes beyond the SnO_2_ materials for lithium ion batteries.

## Methods

### Synthesis of SnO_2_ octahedrons

SnO_2_ octahedrons were prepared via a hydrothermal method^39^. Typically, SnCl_4_·5H_2_O (2 mmol), HCl (36.5%, 1.2 mL) and poly(vinyl pyrrolidone) (PVP, 0.012 mmol) were sequentially dispersed into ethanol/ultrapure water (12 mL, 1/1 v/v) under intense sonication. The resulting solution was transferred to a Teflon-lined stainless steel autoclave (50 mL) and maintained at 200 °C for 12 h. The obtained products were collected after being washed with ultrapure water and ethanol for several times.

### Preparation of GCNT-SnO_2_ aerogels

CNTs were purchased from Sigma-Aldrich (30~50 nm, ~10 μm in length). Graphene oxide (GO) was prepared according to a modified Hummers’ method^44^. Pristine CNTs (100 mg) with bundle morphology can be uniformly dispersed by GO solution (150 mL, 2 mg/mL) under sonication^44^. SnO_2_ octahedrons (300 mg, 530 mg, 1030 mg and 1600 mg) were dispersed into the GO/CNT suspension with the assistance of 2-[2-(2-methoxyethoxy)ethoxy]acetic acid (MEEAA), and the mixed materials were washed by ultrapure water for several times to remove the additional MEEAA. And a little amount (0.5 wt%) of PAA was added into the above hybrid solution with assistance of triethylamine. Then, the prepared composite solution was directionally frozen by dipping into liquid nitrogen under a constant speed of 2 mm/min. Freeze-drying treatment (less than 30 Pa) was utilized to completely remove the ice pillars meanwhile retain the aligned pores inside the product. The obtained bulk hybrid materials were then treated with pyrolysis at 350 °C for 2 h in air and then at 800 °C for 2 h in Ar, resulting in the formation of GCNT-SnO_2_(1), GCNT-SnO_2_(2), GCNT-SnO_2_(3) and GCNT-SnO_2_(4) aerogels. The residual oxygen containing groups introduced on the surface of GO sheets were removed under high temperature pyrolysis. The ultrathin PAA film was conducted with imidization and carbonization treatments under 350 °C and 800 °C, achieving the formation of ultrathin carbonic film on the surface of GCNT-SnO_2_ aerogel.

### Characterizations

The structures and morphologies of the samples were studied with a field-emission SEM (Hitachi S-4800). EDS was conducted on an Oxford instrument (X-Max 50). XRD patterns were conducted on a Bruker D8 GADDS X-ray diffractometer with Cu Kα radiation. TEM and HRTEM investigations were carried out with a Tecnai G2 F20 microscope (FEI). Nitrogen adsorption/desorption isotherms were measured on an Auto sorb-1 Quantachrome Instruments at 77 K. TGA was conducted in air at a heating rate of 5 °C/min. XPS measurements were carried out on a Thermo ESCALAB 250Xi spectrometer with an Al Kα X-ray source (1486.6 eV), X-ray radiation (15 kV and 10 mA) and hemispherical electron energy analyzer.

### Electrochemical measurements

The electrochemical tests were performed in a two electrode system of columnar mold, in which the aerogel can be directly used as cathode electrode without any pressure or extrusion, and pure lithium foils were used as counter and reference electrodes. Here, GCNT-SnO_2_ aerogels were used as working electrode without any binder and current collector. 1 M LiPF6 in ethylene carbonate-dimethyl carbonate-diethyl carbonate (1:1:1, weight percent) was taken as electrolyte. Celgard 2400 microporous polypropylene membrane was used as a separator. The LIBs were assembled in an Ar filled glovebox with oxygen and water contents of less than 1 ppm. Cyclic voltammograms were recorded from 0 to 2.5 V on ARBIN electrochemical working station (MSTAT-10 V/10 mA/48Ch) at a scan rate of 0.1 mV/s. Charge/discharge curves, rate performance, and long-term cycling tests were recorded on LAND 2001 A testing systems. Electrochemical impedance measurements were carried out on a Solartron electrochemical interface analysis system (SI 1260, SI 1287). And the Nyquist plots were recorded potentiostatically by applying an AC voltage of 10 mV from 100 KHz to 0.01 Hz.

## Additional Information

**How to cite this article**: Liu, M. *et al*. Octahedral Tin Dioxide Nanocrystals Anchored on Vertically Aligned Carbon Aerogels as High Capacity Anode Materials for Lithium-Ion Batteries. *Sci. Rep.* **6**, 31496; doi: 10.1038/srep31496 (2016).

## Supplementary Material

Supplementary Information

## Figures and Tables

**Figure 1:**
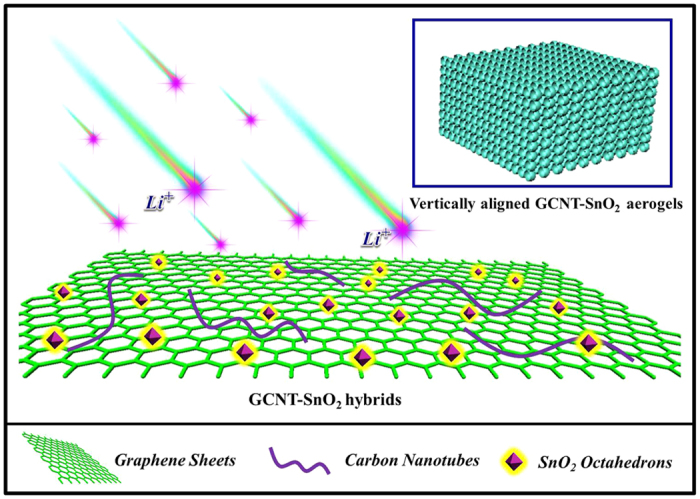
Schematic illustration of GCNT-SnO_2_ aerogels with vertically aligned pores, and the rapid insertion/extraction of lithium ions.
