# Supplementary material for: Octahedral Tin Dioxide Nanocrystals Anchored on Vertically Aligned Carbon Aerogels as High Capacity Anode Materials for Lithium-Ion Batteries
Source: Sci Rep. 2016 Aug 11;6:31496. doi: 10.1038/srep31496 (PMC4980600; doi:10.1038/srep31496)
Supplement: Supplementary Information [file srep31496-s1.doc]

Electronic Supplementary Data

**Octahedral Tin Dioxide Nanocrystals Anchored on Vertically Aligned Carbon Aerogels as High Capacity Anode Materials for Lithium-Ion Batteries**

Mingkai Liu^1,3^, Yuqing Liu^1^, Yuting Zhang^1^, Yiliao Li^1^, Peng Zhang^1^, Yan Yan^1,*^, Tianxi Liu^1,2,*^

^1^School of Chemistry and Chemical Engineering, Jiangsu Key Laboratory of Green Synthetic Chemistry for Functional Materials, Jiangsu Normal University, Xuzhou 221116, China.

^2^State Key Laboratory for Modification of Chemical Fibers and Polymer Materials, College of Materials Science and Engineering, Donghua University, Shanghai 201620, China.

^3^State Key Laboratory of Molecular Engineering of Polymers, Department of Macromolecular Science, Fudan University, Shanghai 200438, China.

Corresponding authors. E-mail addresses: [yanyan@jsnu.edu.cn](mailto:yanyan@jsnu.edu.cn), [txliu@fudan.edu.cn](mailto:txliu@fudan.edu.cn)


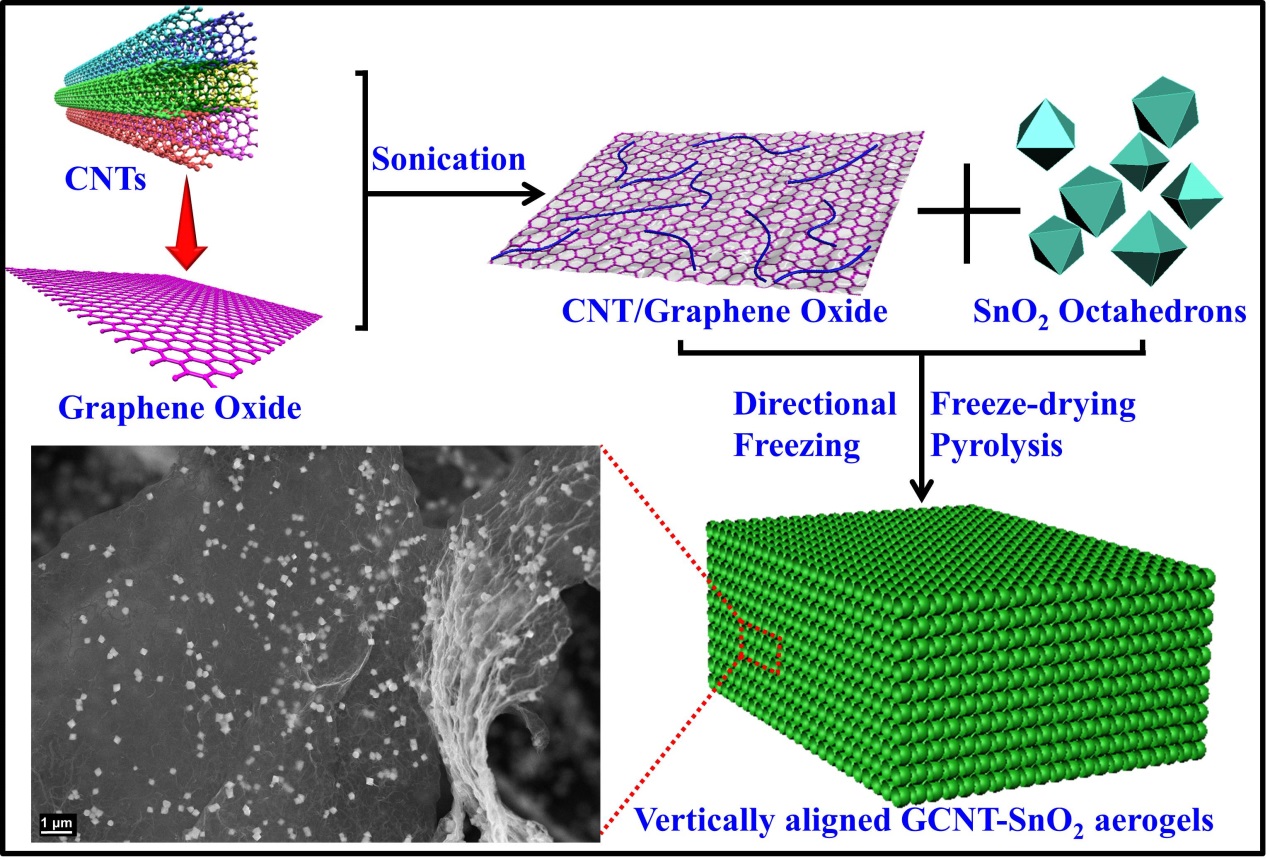


**Figure S1.** Schematic illustration of the fabrication process of GCNT-SnO_2_ aerogels with vertically aligned pores.


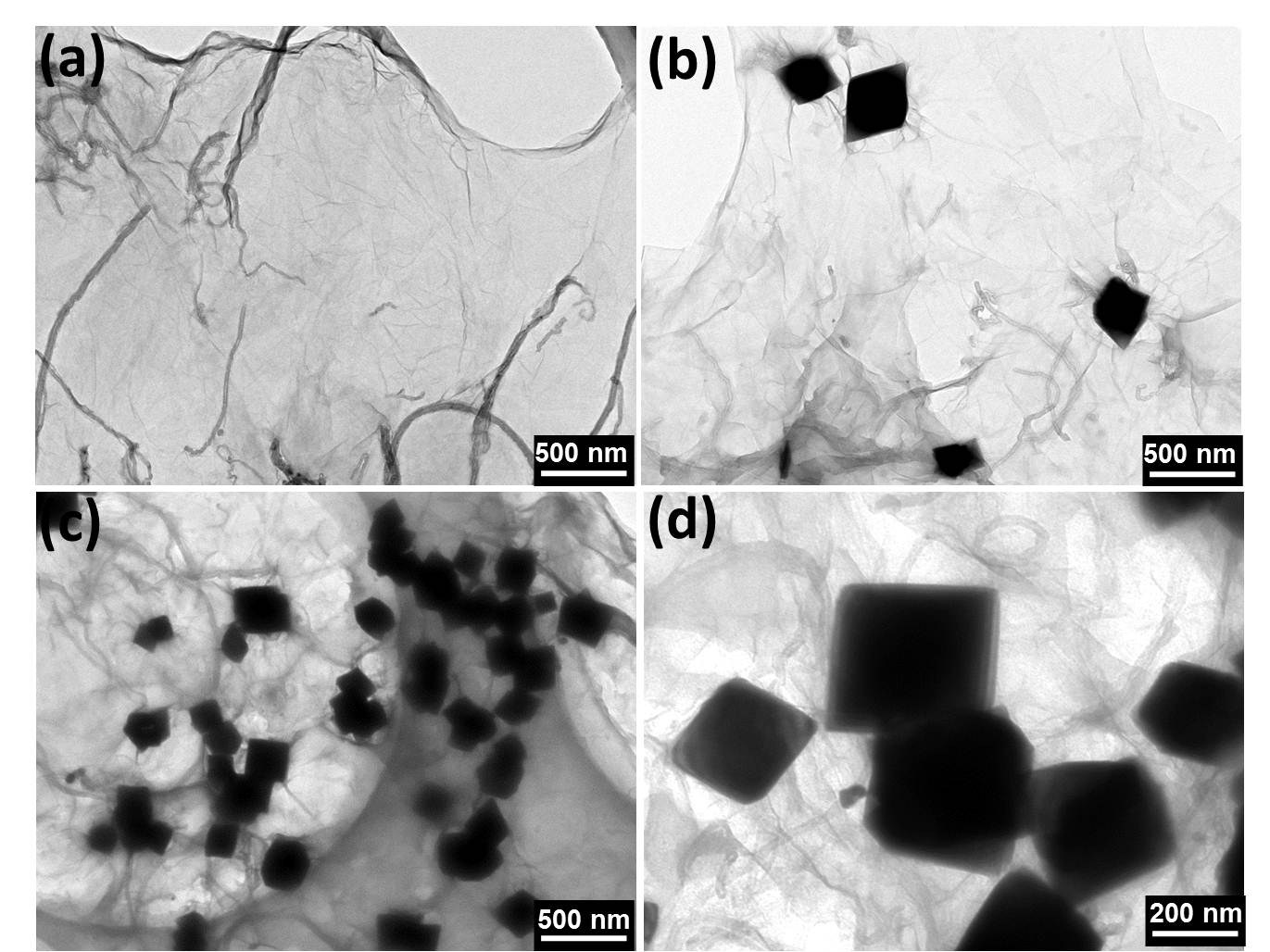


**Figure S2.** TEM images of (a) GCNT, (b) GCNT-SnO_2_(1), (c and d) GCNT-SnO_2_(3) at low and high magnifications.


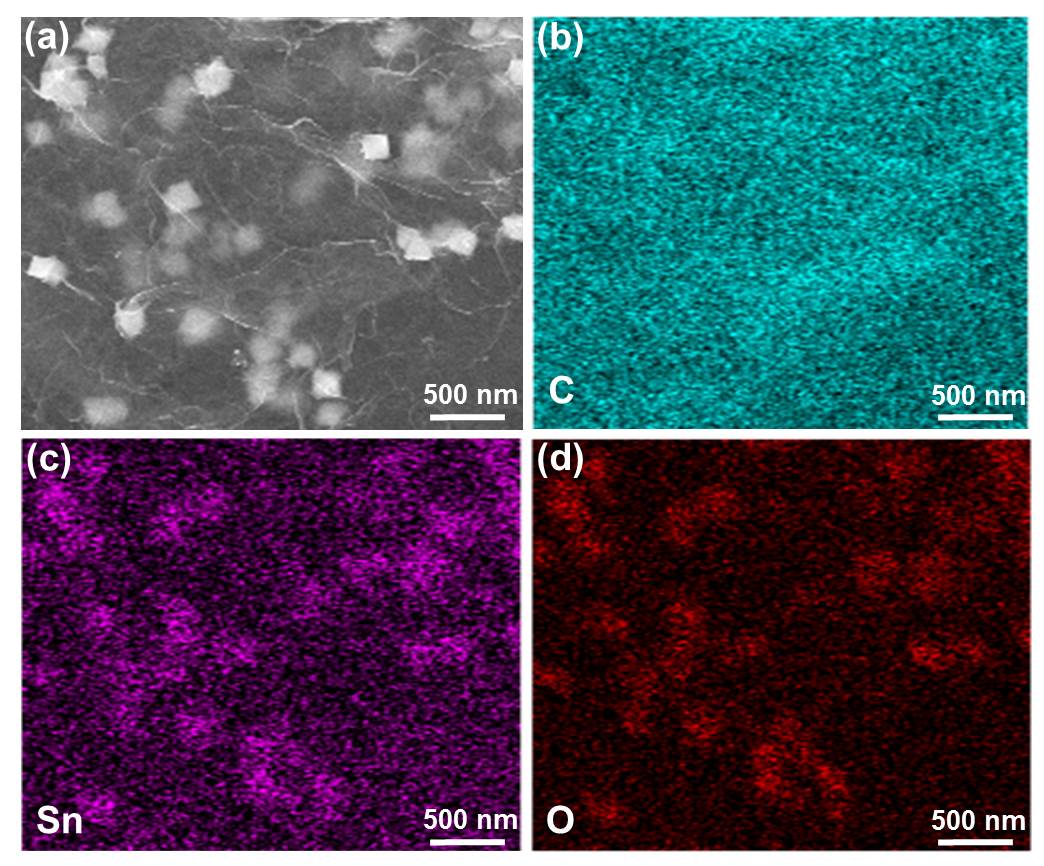


**Figure S3.** SEM image of (a) GCNT-SnO_2_(3) aerogel and the corresponding EDS mapping of (b) C, (c) Sn, and (d) O elements.


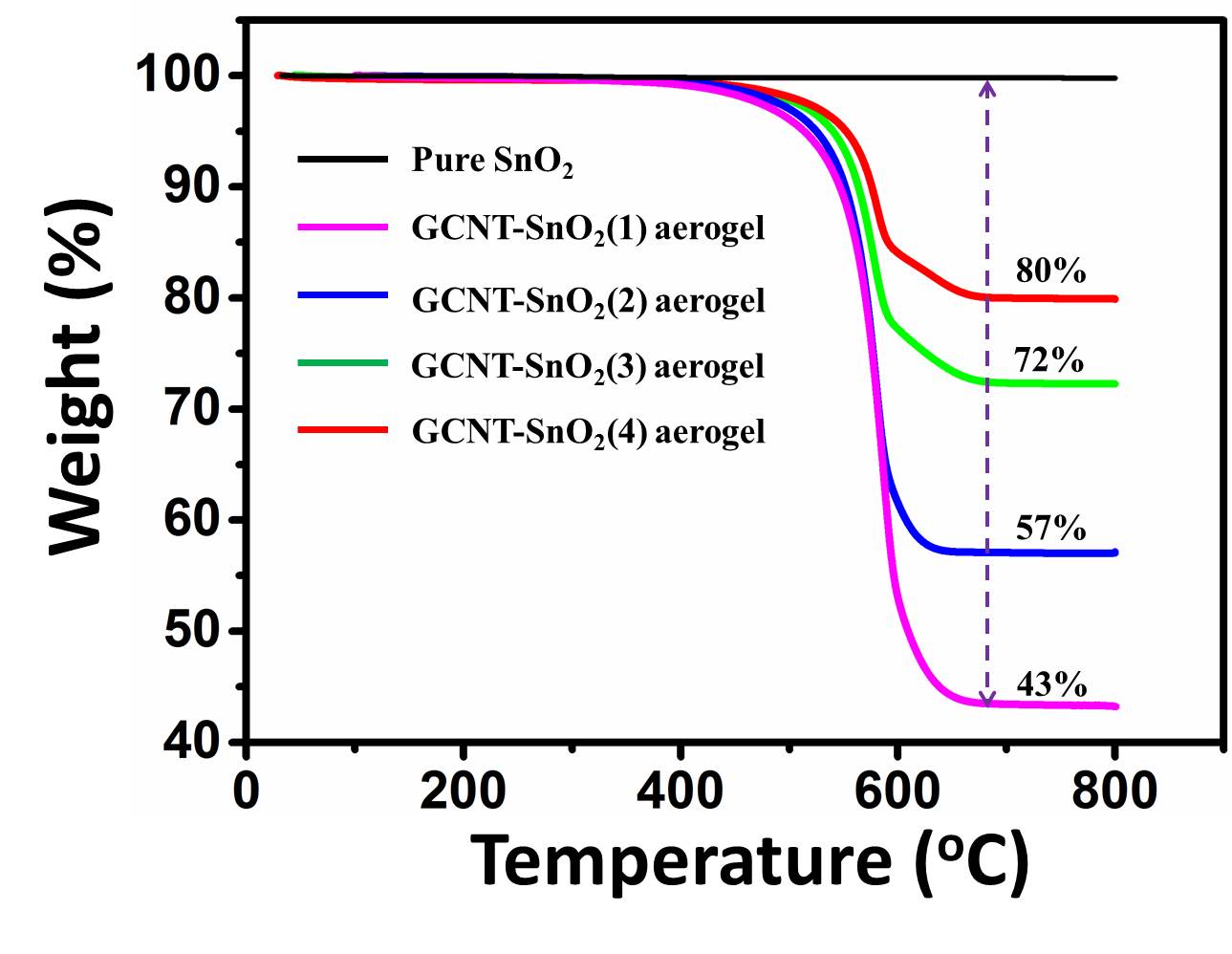


**Figure S4.** TGA curves of pure SnO_2_ octahedron and GCNT-SnO_2_ aerogels.


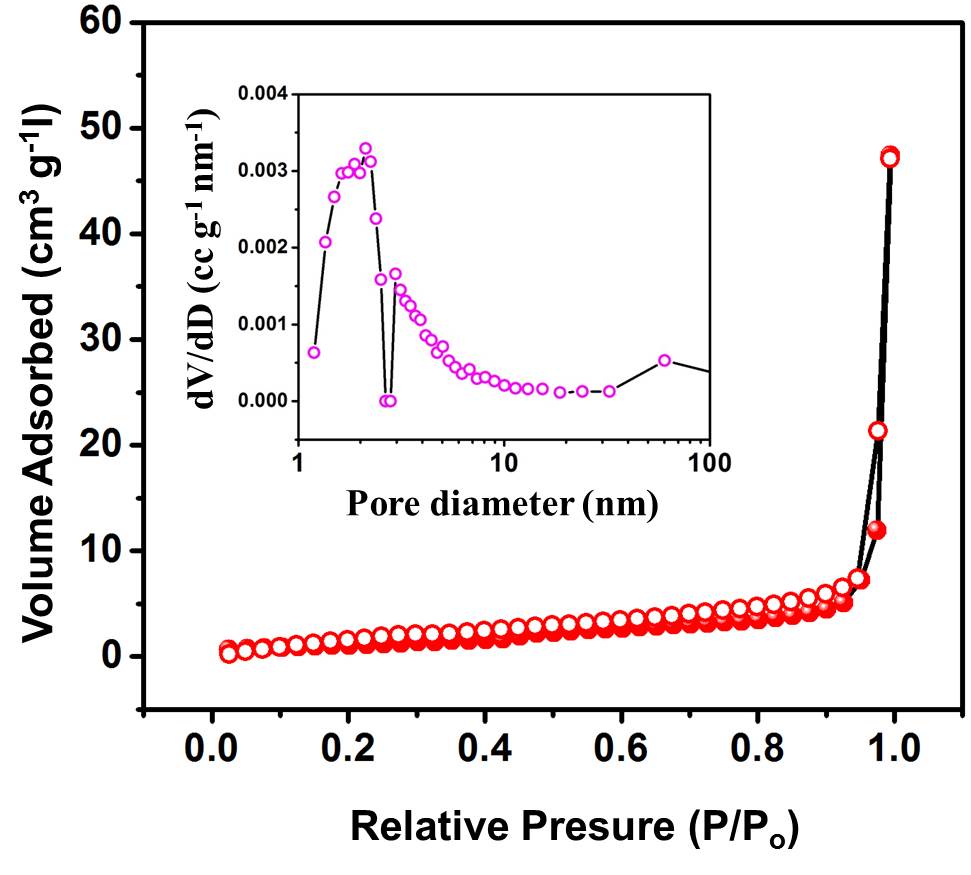


**Figure S5.** Nitrogen adsorption/desorption isotherm and the corresponding pore size distribution of pure SnO_2_ octahedrons obtained at 77K.


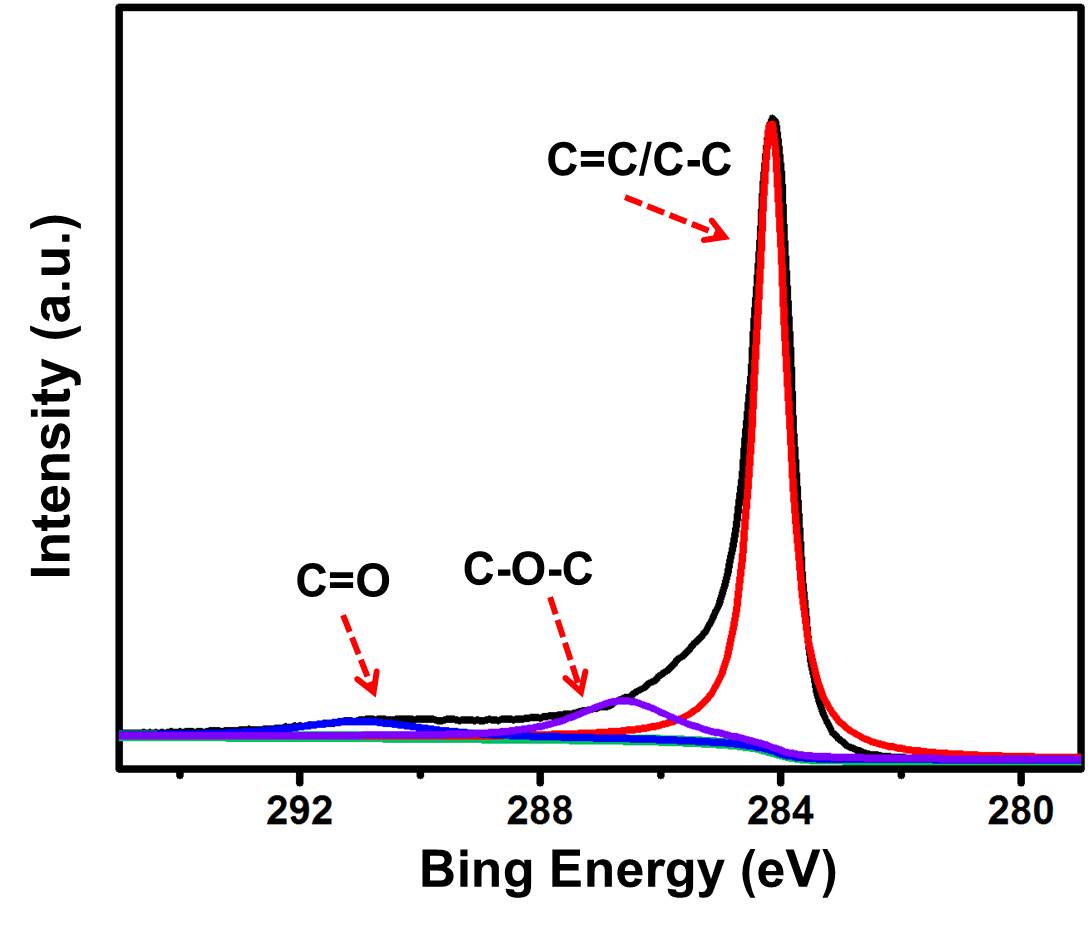


**Figure S6.** High resolution XPS spectra of C 1s region for GCNT-SnO_2_(3) aerogel.


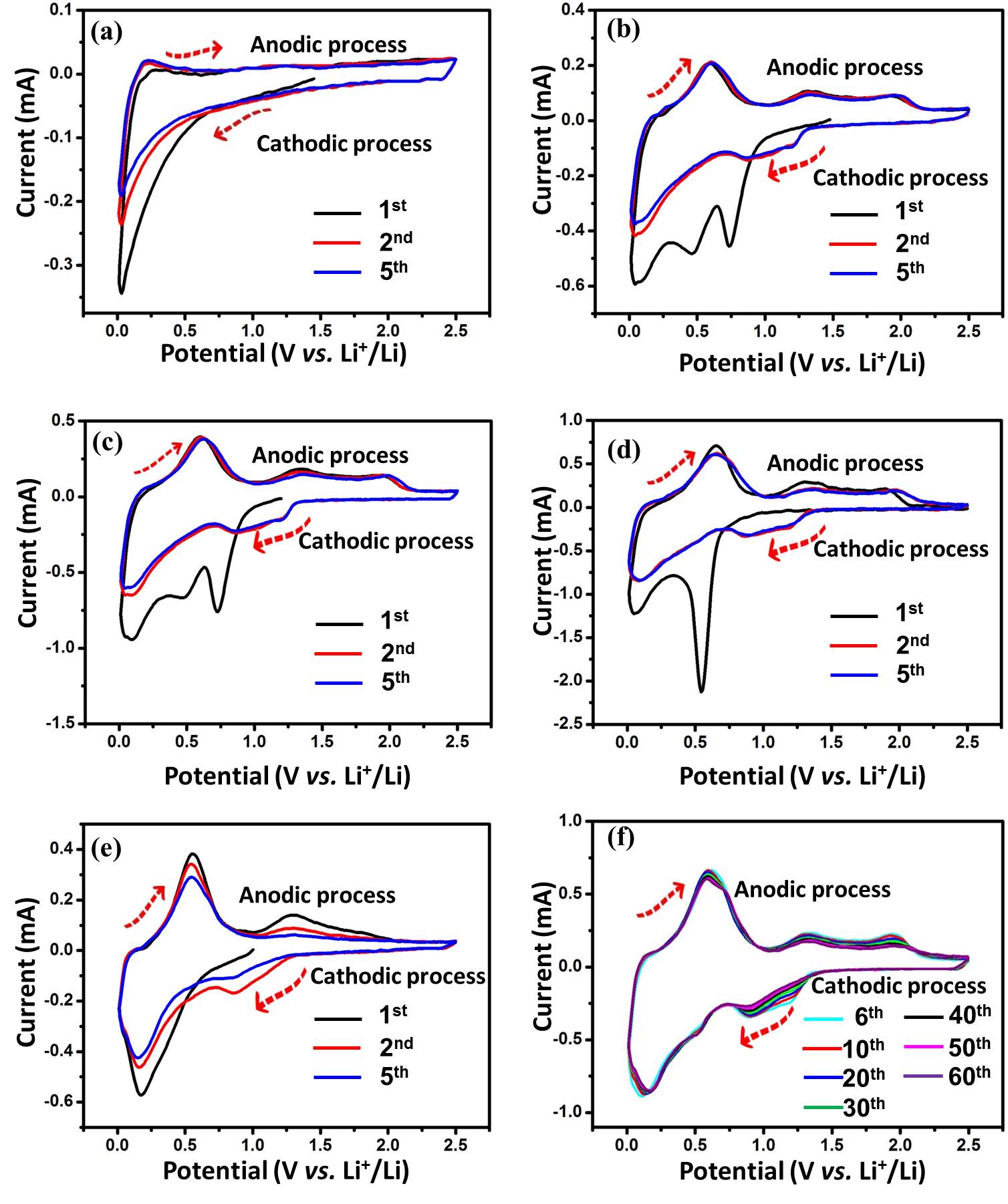


**Figure S7.** CV curves of (a) GCNT, (b) GCNT-SnO_2_(1) aerogel, (c) GCNT-SnO_2_(2) aerogel, (d) GCNT-SnO_2_(4) aerogel, (e) pure SnO_2_ octahedrons at 0.1 mV/s at the 1^st^, 2^nd^, and 5^th^ cycle, and (f) CV curves of GCNT-SnO_2_(3) aerogel at different cycles from 6^th^ to 60^th^.


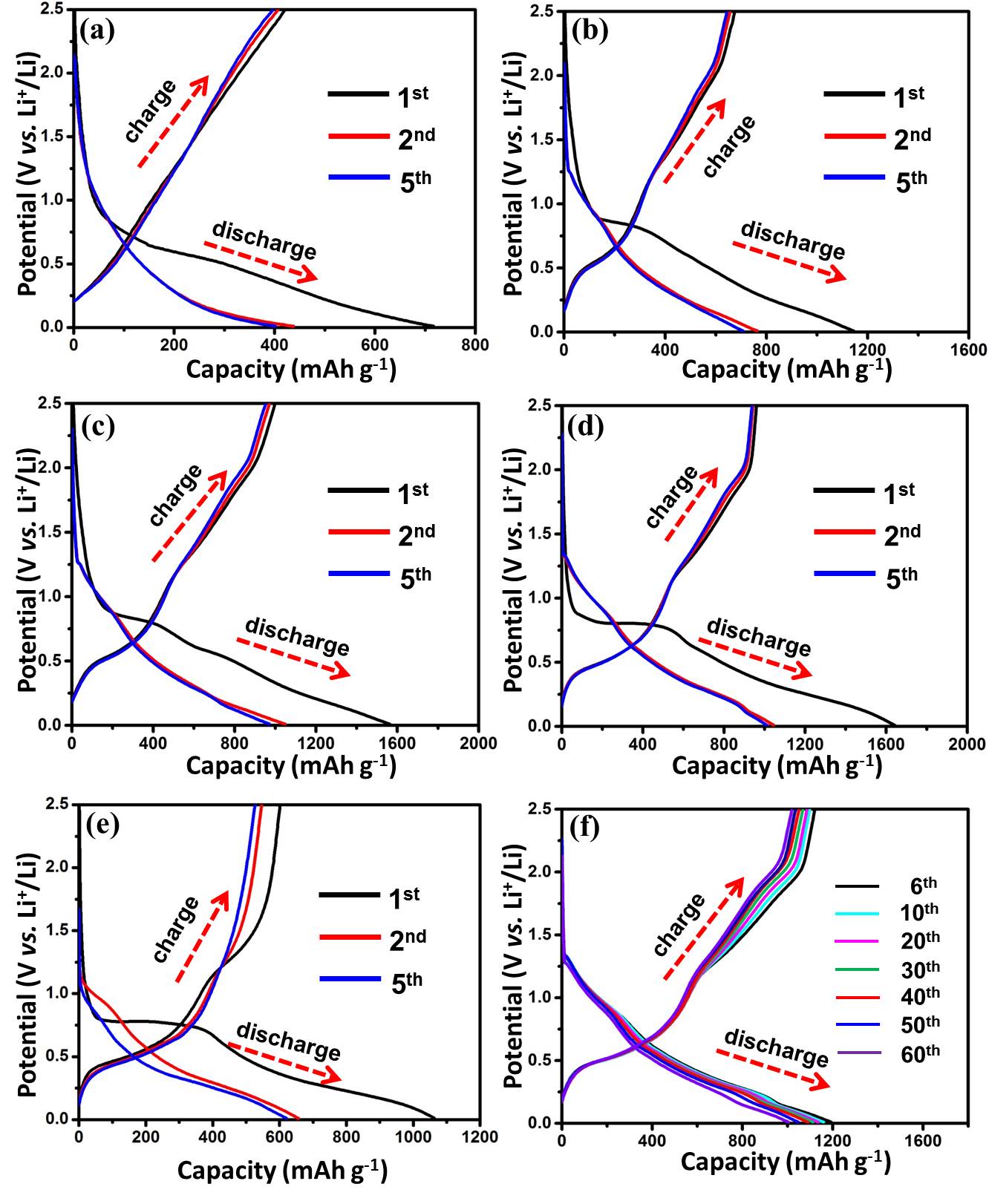


**Figure S8.** The 1^st^, 2^nd^, and 5^th^ charge/discharge curves of (a) GCNT, (b) GCNT-SnO_2_(1) aerogel, (c) GCNT-SnO_2_(2) aerogel, (d) GCNT-SnO_2_(4) aerogel, (e) pure SnO_2_ octahedrons at 0.1 A/g, and (f) charge/discharge curves of GCNT-SnO_2_(3) aerogel at different cycles from 6^th^ to 60^th^.


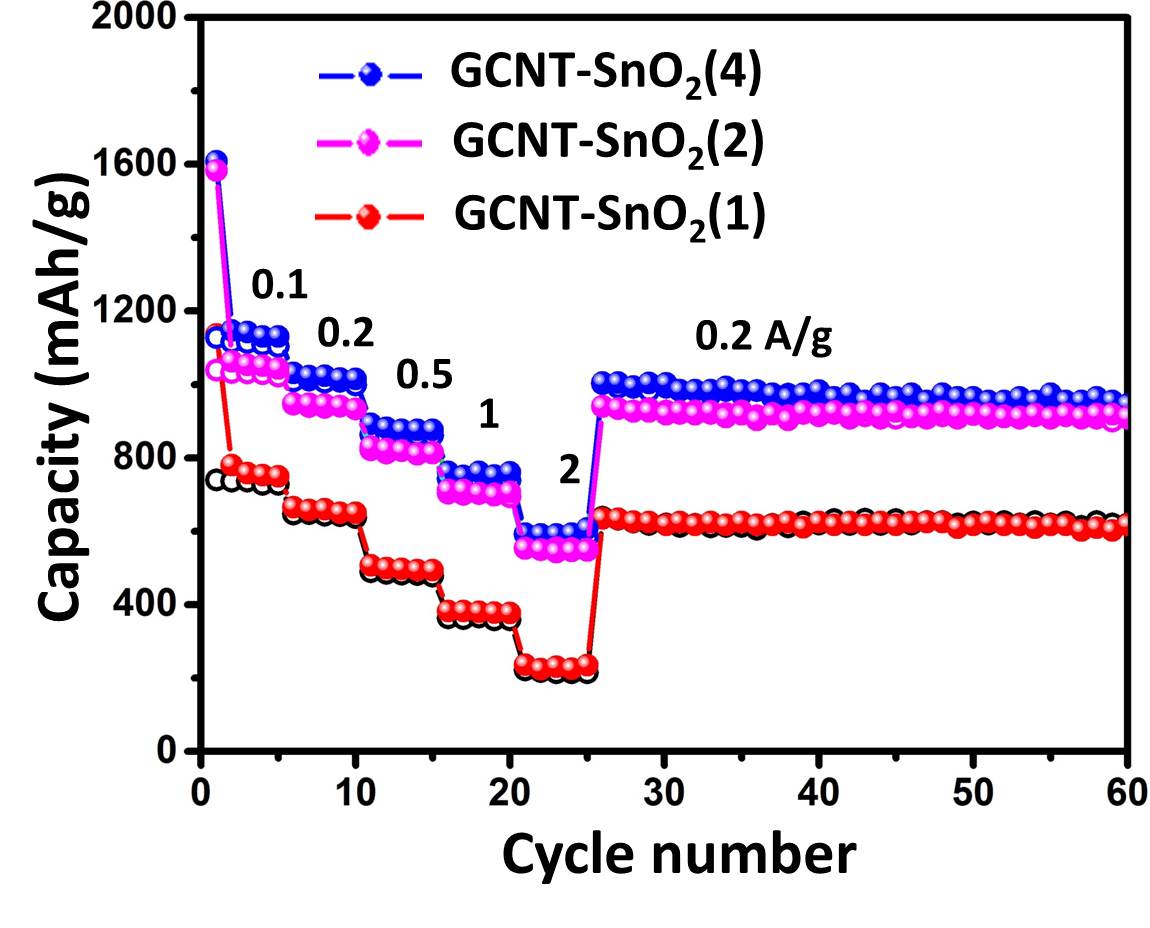


**Figure S9.** Rate performance of GCNT-SnO_2_(1), GCNT-SnO_2_(2), GCNT-SnO_2_(4) aerogels at various current rates from 0.1 to 2 A/g.


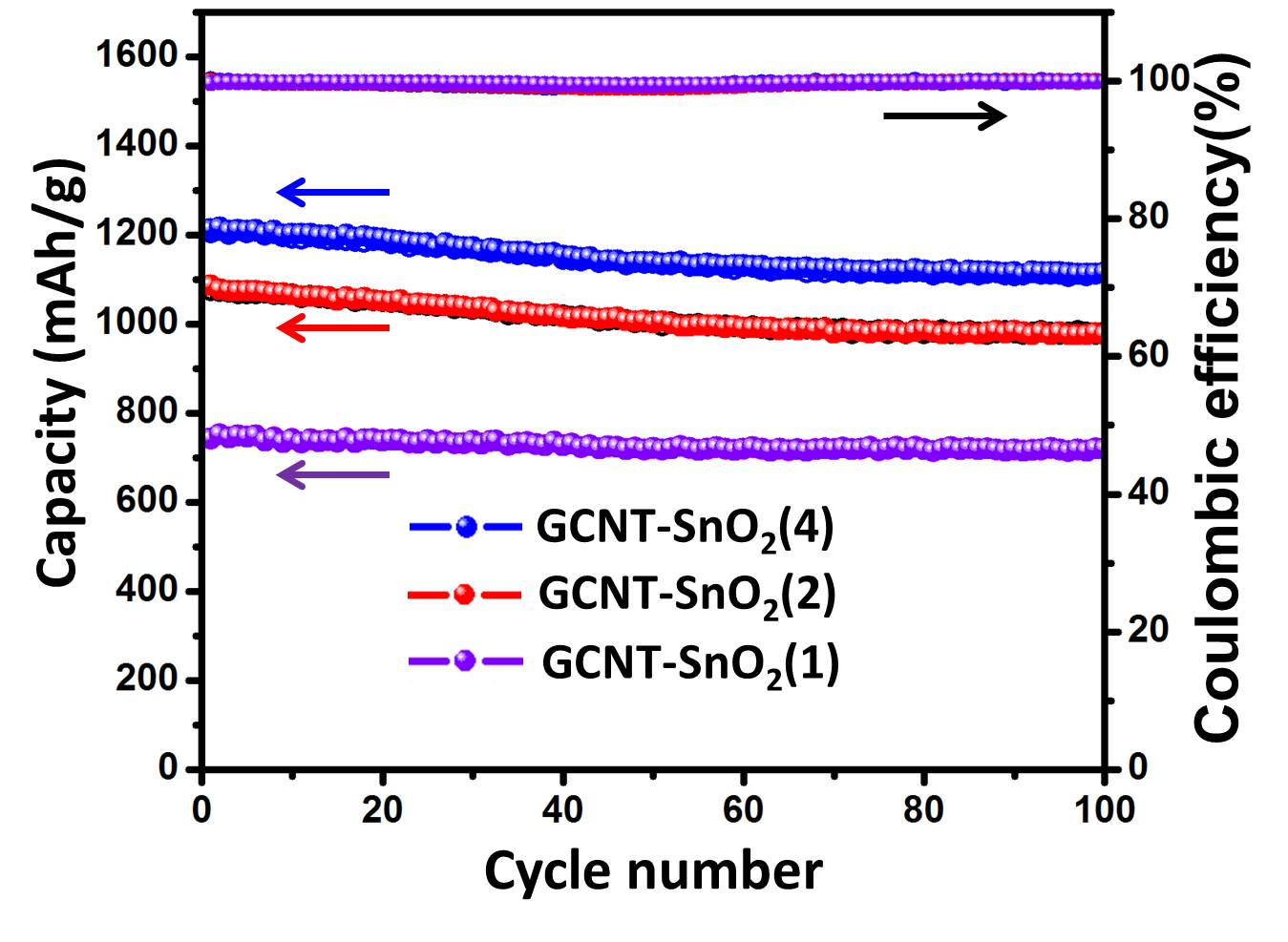


**Figure S10.** Cycling performance of GCNT-SnO_2_(1), GCNT-SnO_2_(2), GCNT-SnO_2_(4) aerogels at 0.1 A/g, coupled with their corresponding Coulombic efficiencies.


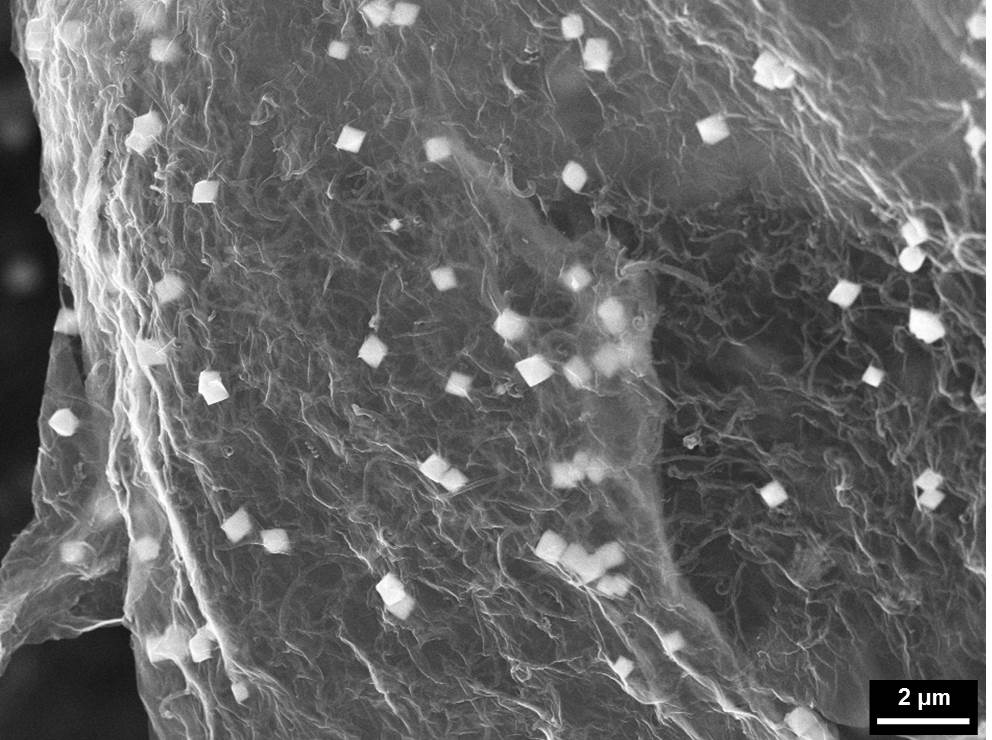


**Figure S11**. SEM image of GCNT-SnO_2_(3) aerogel after the 1^st^ cycle.


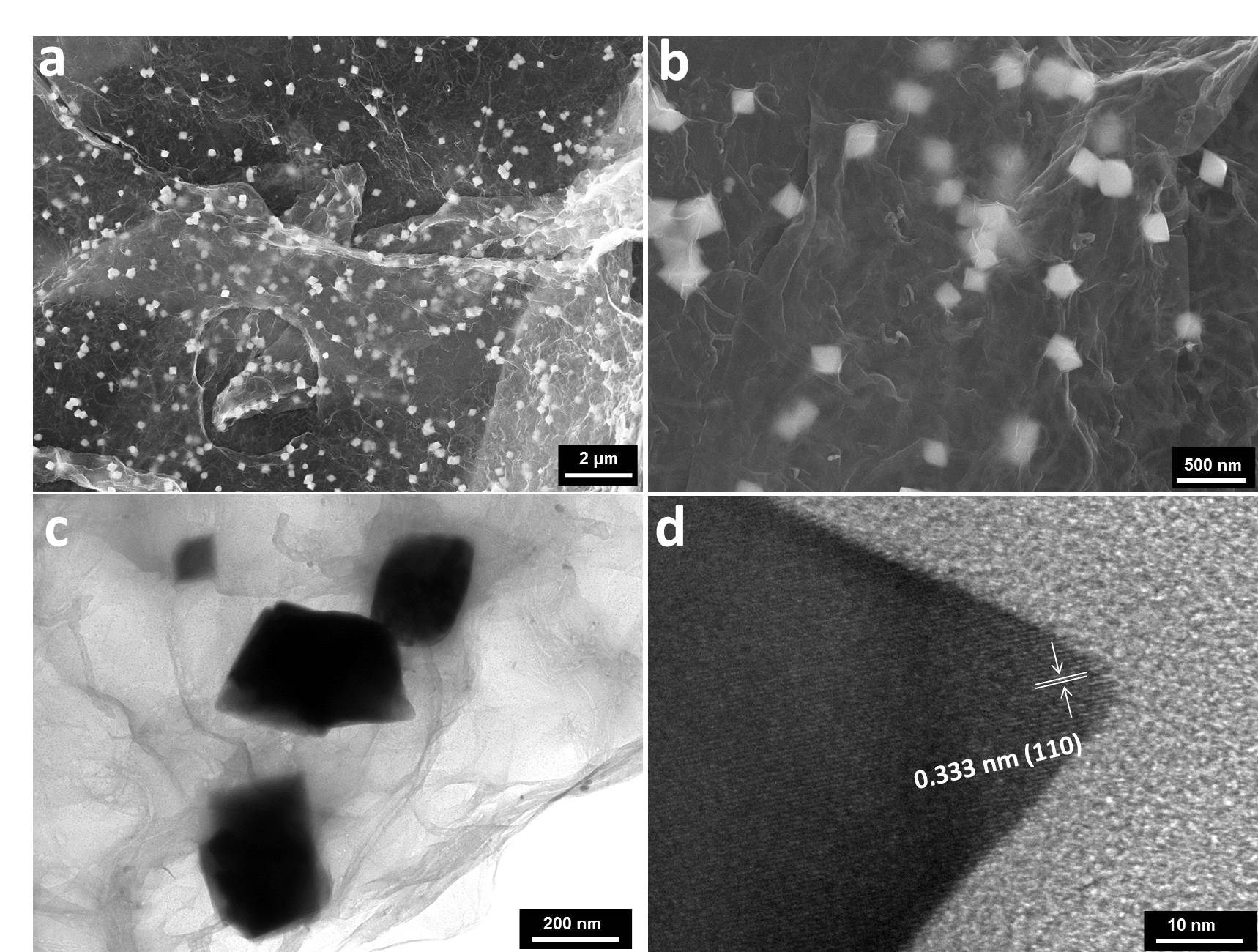


**Figure S12**. (a, b) SEM images of GCNT-SnO_2_(3) aerogel at low and high magnifications after the 100^th^ cycles. (c) TEM image of the GCNT-SnO_2_(3) aerogel after the 100^th^ cycles, and (d) HRTEM image of single SnO_2_ octahedron.


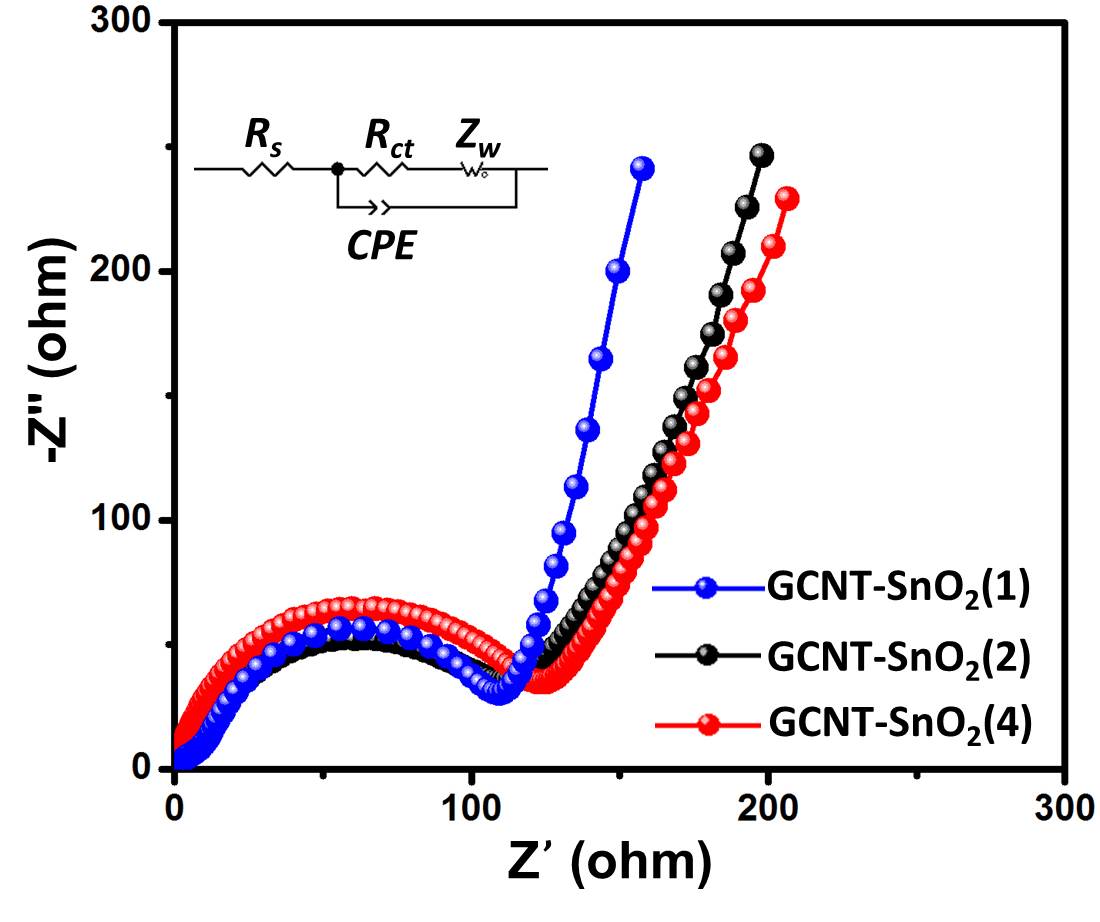


**Figure S13.** Nyquist plots of GCNT-SnO_2_(1), GCNT-SnO_2_(2), GCNT-SnO_2_(4) aerogels.


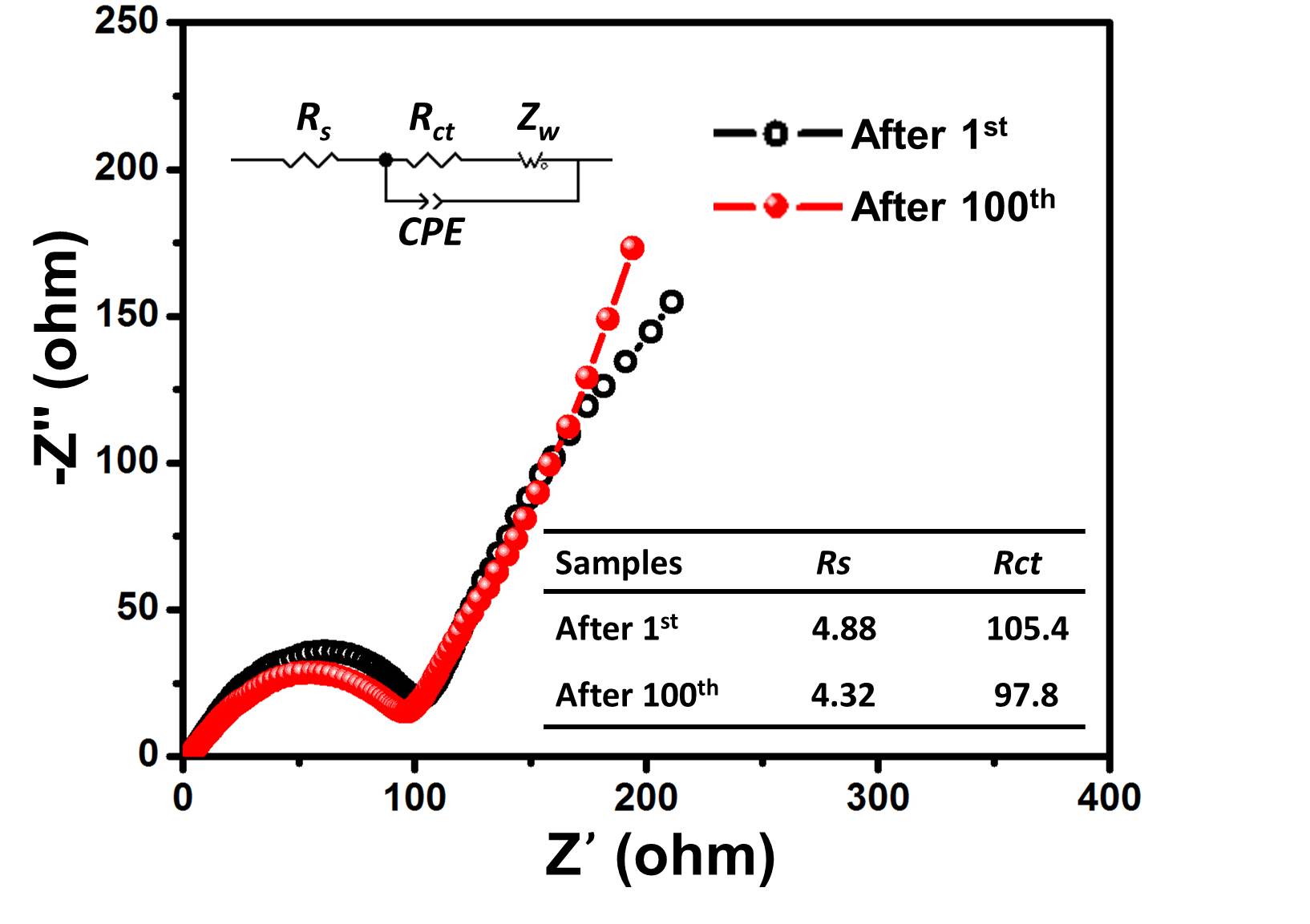


**Figure S14.** Nyquist plots of GCNT-SnO_2_(3) aerogel electrode after being cycled for 1 and 100 times.

**Table S1.** Sheet resistance and resistivity through the thickness direction (ρ_T_) of GCNT, GCNT-SnO_2_ aerogels, and pure SnO_2_ octahedrons.

**Samples Sheet resistance (****Ω sq^-1^)** **ρ_T_ (10^4^ Ω cm)**

**GCNT 50.3 9.2**

**GCNT-SnO_2_(1) 79.4 14.3**

**GCNT-SnO_2_(2) 88.4 16.4**

**GCNT-SnO_2_(3) 89.7 17.1**

**GCNT-SnO_2_(4) 105.9 25.2**

**SnO_2_ Octahedron 323.7 /**

**Table S2.** Resistance values of GCNT, GCNT-SnO_2_ aerogels and pure SnO_2_ octahedrons calculated according to the equivalent circuit mode.

**Samples *R*_s_ *R*_ct_ *Z*_w_ *CPE***

**GCNT 3.01 55.6 0.781 0.556**

**GCNT-SnO_2_(1) 3.87 101.3 0.851 0.633**

**GCNT-SnO_2_(2) 4.23 109.8 0.916 0.666**

**GCNT-SnO_2_(3) 4.88 105.4 0.898 0.685**

**GCNT-SnO_2_(4) 4.95 119.5 0.986 0.701**

**SnO_2_ Octahedrons 5.64 264.8 1.008 0.799**
